# The clinical features and treatment modality of esophageal neuroendocrine tumors: a multicenter study in Korea

**DOI:** 10.1186/1471-2407-14-569

**Published:** 2014-08-07

**Authors:** Chang Geun Lee, Yun Jeong Lim, Seun Ja Park, Byung Ik Jang, Seok Reyol Choi, Jae Kwang Kim, Yong-Tae Kim, Joo Young Cho, Chang Hun Yang, Hoon Jai Chun, Si Young Song, Neuroendocrine tumor study group

**Affiliations:** Department of Internal Medicine, Dongguk University Ilsan Hospital, Dongguk University-Seoul, Graduate School of Medicine, Goyang, Korea; Department of Internal Medicine, Kosin University College of Medicine, Busan, Korea; Department of Internal Medicine, Yeungnam University School of Medicine, Daegu, Korea; Department of Internal Medicine, Busan Seong-So Hospital, Busan, Korea; Department of Internal Medicine, Catholic University of Korea, College of Medicine, Seoul, Korea; Department of Internal Medicine, Seoul National University College of Medicine, Seoul, Korea; Department of Internal Medicine, Soonchunhyang University College of Medicine, Seoul, Korea; Department of Internal Medicine, Korea University College of Medicine, Seoul, Korea; Department of Internal Medicine, Yonsei University College of Medicine, Seoul, Korea

**Keywords:** Esophagus, Neuroendocrine tumor, Treatment, Prognosis

## Abstract

**Background:**

Neuroendocrine tumors (NETs) of the esophagus are extremely rare, and few cases have been reported worldwide. Thus, a comprehensive nationwide study is needed to understand the characteristics of and treatment strategy for esophageal NETs.

**Methods:**

We collected data on esophageal NET patients from 25 hospitals in Korea from 2002–2012. The incidence, location, clinical symptoms, histopathology, treatment response, and the biochemical, radiologic and endoscopic characteristics of esophageal NETs were surveyed.

**Results:**

Among 2,037 NETs arising in different gastrointestinal sites, esophageal NETs were found in 26 cases (1.3%). The mean patient age was 60.12 ± 9.30 years with a 4:1 male predominance. In endoscopic findings, 76.9% (20/26) of NETs were located in the lower third of the esophagus and the mean size was 2.34 ± 1.63 cm. At diagnosis, more than half the patients (15/26, 57.7%) had regional lymph node metastasis or widespread metastasis. Endoscopic resection was conducted in three cases, and in all three of them, lymph node metastasis was not found and tumor size was below 1.0 cm. All tumors were completely removable through endoscopic procedures and there was no recurrence during the follow-up period. Eighteen other patients received an operation, chemotherapy or both. Among them, nine patients (50.0%) expired because of the progression of their cancer or post-operative complications. In Kaplan-Meier survival analysis, only tumor size (more than 2.0 cm) showed prognostic significance (*P* = 0.045).

**Conclusions:**

Despite the general assumption that gastrointestinal NETs are benign and slow-growing tumors, the prognosis of advanced esophageal NETs is not favorable.

## Background

Gastrointestinal (GI) neuroendocrine tumors (NETs) are relatively rare, but their incidence has been sharply rising in recent decades, and there have recently been several studies on GI NETs [1–3]. According to the organ distribution and frequency, pathogenesis and treatment modality can differ in terms of prognosis; therefore, it is important to understand the characteristics of each organ through observation [1, 4]. However, NETs in the esophagus are exceedingly rare, and there have been no concrete data published on clinical features or prognosis [5–7].

Few cases concerning primary esophageal NETs have been reported in the literature. This is because the neuroendocrine system is not well developed in the esophagus [5]. Previously, one study reported on 8,305 NETs at different anatomical sites; however, only three (0.04%) were reported to be esophageal NETs [8]. In the latest multicenter research conducted in Korea in 2012, 4,951 cases of gastroenteropancreatic NETs were analyzed, and of these, only 1.4% were reported to be esophageal NETs [1].

Because of the paucity of data, the incidence, clinical features of and treatment strategies for esophageal NETs have not yet been defined. Moreover, there are no studies describing a definitive treatment strategy or prognosis associated with primary esophageal NETs. Thus, this multicenter study was undertaken to assess the incidence, clinical characteristics, treatment modality and prognosis of esophageal NETs. Here, we describe the incidence, clinicopathologic features, immunohistochemical findings, treatment modality and prognosis of 26 cases of primary esophageal NETs that were treated at various centers over a 10-year period (2002–2012).

## Methods

### Data collection

We collected and reviewed cases of patients diagnosed with primary esophageal NETs from 2002–2012 at 25 universities and general hospitals in Korea. Inclusion criteria were all pathologically confirmed NETs of the esophagus, regardless of the quality of the pathologic reports or histologic classification. Exclusion criteria included the followings: (1) clinical data were not available even if the pathologic report proved it was an esophageal NET; and (2) the patient had a history of neuroendocrine carcinoma elsewhere. Patient age, gender, presenting symptoms, methods of tumor diagnosis, tumor size, multiplicity, mitotic count (about 10 high power fields), Ki-67 labeling index, immunohistochemical expression of synaptophysin and chromogranin, lymphovascular invasion, perineural invasion, lymph node metastasis, radiologic findings, types of treatment and response to treatment were all surveyed.

The location of the tumor in the esophagus was determined on the basis of endoscopic findings and was divided into three segments: an upper (15–24 cm from the incisor teeth), a middle (24–32 cm from the incisor teeth) and a lower (32–40 cm from the incisor teeth) one.

Cases had been clinically evaluated via endoscopic ultrasonography (EUS), computed tomography (CT) and positron emission tomography (PET). Regional LN metastasis or distant metastasis was mainly evaluated using imaging modalities including CT, EUS as well as other modalities. If metastasis was uncertain, a biopsy was performed at the suspected site. If an operation had been performed, metastasis was confirmed by a post-operative pathologic report. Neuroendocrine differentiation was confirmed using immunohistochemical staining for synaptophysin and chromogranin. Retrospective collection of gastroenteropancreatic NETs had been approved by each Institutional Review Board of all participating centers (“Busan National University” Hospital, “Catholic University of Korea Seoul Saint” Hospital, “Chonnam National University” Hospital, “Chungnam National University” Hospital, Daegu Catholic University Medical Center, “Dong-A University” Hospital, “Dongguk University Ilsan” Hospital, “Eulji General” Hospital, Gachon University Gil Medical Center, “Gangwon National University” Hospital, “Gyeongsang National University” Hospital, Hanyang University Medical Center, “Inha University” Hospital, “Inje University Haeundae Paik” Hospital, “Inje University Sanggye Paik” Hospital, Konkuk University Medical Center, Korea University Medical Center, “Kosin University Gospel” Hospital, National Cancer Center, “Seoul National University Bundang” Hospital, “Seoul National University” Hospital, “Soonchunhyang University” Hospital, Yeungnam University Medical Center, “Yonsei University Kangnam Severance” Hospital, “Yonsei University Sinchon Severance” Hospital).

### Staging and classification

If data on tumor size and extension were available, we also classified tumors according to the 2000 WHO classification criteria into; well-differentiated endocrine tumors (WDETs), well-differentiated endocrine carcinomas (WDECs), poorly differentiated endocrine carcinomas/small cell carcinomas (PDECs), mixed exocrine-endocrine carcinomas (MEECs), and metastatic endocrine carcinomas (MECs) [9]. In addition, the 2010 WHO classification was performed based on the grading of the mitotic or Ki-67 labeling index [9]. Mitosis was recorded as G1 (<2/10 HPF), G2 (2–20/10 HPF) or G3 (>20/10 HPF). The Ki-67-labeling index was recorded as G1 (≤2%), G2 (3–20%) or G3 (>20%). Results of immunohistochemical staining, lymphovascular invasion, perineural invasion and lymph node metastasis were presented as either positive or negative.

### Survival outcome

Survival outcome data were derived through clinical chart review. Overall survival was calculated from the day of esophagogastroduodenoscopy and biopsy to the date of death or last follow-up (months).

### Statistical analysis

Statistical analysis was performed using SPSS software (SPSS Corp, Chicago, IL, USA). Data were represented as the mean ± standard deviation for continuous variables or number (%) for categorical data. To estimate the association between eligible variables and mean survival time, Kaplan-Meier analysis was applied. A *P*-value less than 0.05 was considered statistically significant.

## Results

### Clinical characteristics

We collected 2,037 pathology reports of patients with gastroenteropancreatic NETs. Among them, 26 cases (1.3%) were detected in the esophagus. The clinical characteristics of the 26 primary esophageal NETs are summarized in Table 1. Mean patient age was 60.12 ± 9.30 (range, 45–76 years) with approximately a 4:1 male predominance.

**Table 1:** **Demographic and clinical characteristics of 26 esophageal neuroendocrine tumors**

	Number (%)
Male sex (%)	21 (80.8)
Mean age (range)	60.12 ± 9.30 (45–76)
Symptoms at diagnosis	
Asymptomatic	8 (30.8)
Dysphagia	7 (26.9)
Abdominal discomfort	5 (19.2)
Weight loss	3 (11.5)
Melena	2 (7.7)
Hot flushes	1 (3.8)
Diarrhea	1 (3.8)

Among the 26 patients, eight lesions (30.8%) were found incidentally during a routine check-up esophagogastroduodenoscopy. The most common presenting symptom was dysphagia (seven patients, 26.9%), and other symptoms at diagnosis included abdominal discomfort (five patients, 19.2%), weight loss (three patients, 11.5%), and melena (two patients, 7.7%). The typical carcinoid symptom was documented in only one patient (3.8%). Most of the patients (21/26, 80.8%) had an ECOG performance status of 0 or 1.

### Endoscopic findings

The endoscopic findings of the 26 cases of primary esophageal NETs are summarized in Table 2. The primary tumor was most frequently located in the lower esophagus (20 patients, 76.2%), followed by the middle esophagus (four patients, 15.4%) and upper esophagus (two patients, 7.7%). The tumor was mainly represented in a single lesion (24/26, 92.3%), and only two cases (7.7%) were found to have multiple lesions. The sizes of the tumors ranged from 0.5–7.5 cm (mean 2.34 ± 1.63 cm; median 1.9 cm). These tumors mostly represented elevated polypoid or nodular elevated types (17/26, 65.4%). The overlying surface showed mostly smooth, glistening and tan-brown discoloration. Some other tumors were represented within the esophageal wall as large infiltrative lesions of elevated and depressed types (6/26, 23.1%) or ulcerated types (3/26, 11.5%).

**Table 2:** **Endoscopic findings of 26 esophageal neuroendocrine tumors**

Endoscopic characteristics	Total (%)
Tumor location	
Upper (15–24 cm)	2 (7.7)
Middle (24–32 cm)	4 (15.4)
Lower (32–40 cm)	20 (76.2)
Tumor lesions	
Single lesion	24 (92.3)
Multiple lesion	2 (7.7)
Tumor size (cm)	
Mean size (range)	2.34 ± 1.63 (0.5–7.5)
0–0.9	4 (15.4)
1.0–1.9	8 (30.8)
2.0–	14 (53.8)
Gross appearance	
Elevated	17 (65.4)
Elevated and depressed	6 (23.1)
Ulcerative	3 (11.5)

### Distribution of NETs according to the 2000 and 2010 WHO classification

The distribution of tumors according to their diagnosis is shown in Table 3. In the classification of tumors according to the 2000 WHO classification, three (11.5%) were well-differentiated endocrine tumors, two (7.7%) were mixed exocrine-endocrine carcinomas and 10 (38.5%) were poorly differentiated endocrine carcinomas. The other 11 patients had no clinical records available, thus the 2000 WHO classification could not be applied. All reported seven esophageal NETs based upon the 2010 WHO classification were categorized as neuroendocrine carcinomas (G3).

**Table 3:** **Pathologic findings of 26 esophageal neuroendocrine tumors**

Clinical characteristics	Number (%)
No. of patients	26 (100)
Tumor differentiation (2000 WHO classification)	
Well differentiated	3 (11.5)
Mixed	2 (7.7)
Poorly differentiated	10 (38.5)
Not mentioned	11 (42.3)
Tumor differentiation (2010 WHO classification)	
G1 (mitotic count <2, Ki-67 < 2)	None
G2 (mitotic count 2–20, Ki-67 3–20)	None
G3 (mitotic count >20, Ki-67 > 20)	7 (26.9)
Not mentioned	19 (73.0)
Tissue immunostaining	
Synaptophysin	23 (88.5)
Chromogranin A	21 (80.8)
CD 56	8 (30.8)

At diagnosis, more than half the patients (15/26, 57.7%) were found to have regional lymph node metastasis or widespread metastasis. Among them, five patients had lymphovascular metastasis and only one patient had a perineural invasion.

Pathologic tissue was collected from esophageal NETs. With regard to tissue immunostaining, 23 out of 26 patients (88.5%) were positive for synaptophysin, and 21 out of 26 patients (80.8%) were positive for chromogranin. However, neither synaptophysin nor chromogranin were detected in any of the serum samples. Urinary 5-hydroxyindoleacetic acid (5-HIAA) levels were also checked, and they were all negative.

Seventeen patients (65.4%) received their final diagnosis through only endoscopic biopsy, while three patients got their final diagnosis only after endoscopic removal of the tumor. Six other patients received their final diagnosis after the operation. There were two cases of esophageal NETs with concurrent squamous cell carcinomas, and one case of an esophageal NET accompanied by an adenocarcinoma.

### Treatment modality

Disease metastasis was assessed using anatomical imaging including CT (15/26, 57.7%), EUS (7/26, 26.9%), ultrasonography of the abdomen (2/26, 7.7%) and PET scans (7/26, 26.9%).

**Figure 1:**
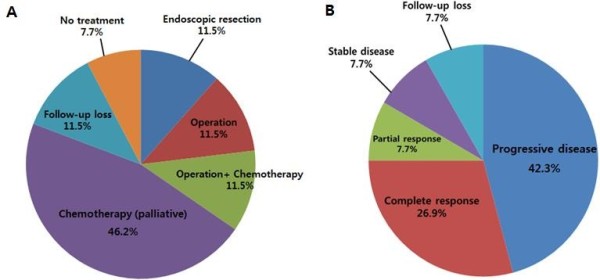
**Treatment modality (A) and treatment response (B) of 26 esophageal neuroendocrine tumors (NETs).** Because of most esophageal NETs were already showing metastasis at the time of diagnosis, prognosis of them did not show benign course. On the other hand, 26.9% of patients showed complete response and all of them were completely removable via endoscopic or surgical methods.

Different treatment modalities and treatment responses are described in Figure 1. Three patients received endoscopic resection only. Their tumor sizes were less than 1.0 cm and were of the elevated type in endoscopic findings. There was no incidence of regional lymph node metastasis, lymphovascular invasion or perineural invasion. All three patients were diagnosed with carcinoid tumor and were alive without tumor recurrence during the follow-up period.

Three patients received surgical resection only. Their tumor sizes were more than 1.0 cm without regional lymph node metastasis, lymphovascular invasion or perineural invasion. In the 2000 WHO classification, all three patients were categorized with poorly differentiated carcinomas. These patients underwent radical resection and esophageal-stomach anastomosis in the thorax or neck. Among them, two cases were alive without recurrence during the follow-up period. However, in one case, a 76-year-old man expired because of post-operative pneumonia.

Three patients received combined modality treatment including surgical resection and chemotherapy. In the 2000 WHO classification, one patient was categorized with a poorly differentiated carcinoma, and two patients were categorized with mixed endocrine-exocrine carcinomas. They received adjuvant systemic chemotherapy, and one patient received additional local radiation therapy. Among them, two patients were alive with progression of the disease, and one patient was alive with stable disease.

**Figure 2:**
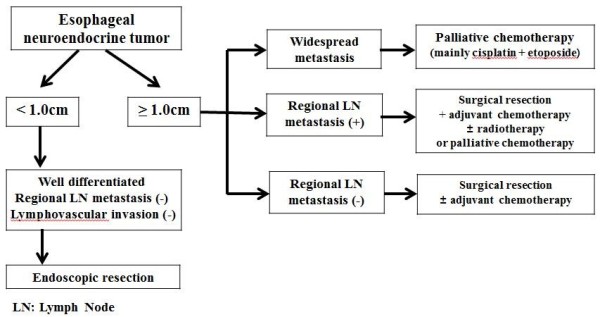
**Treatment algorithm for patients with esophageal neuroendocrine tumors.** It provide treatment strategies that have been organized from these 26 collective cases.

Twelve patients were treated with only palliative chemotherapy. Each patient received different chemotherapy, but most of the regimens began with cisplatin and etoposide (9/12, 75%) as the first combination therapy. When the treatment was found not to be effective, secondary combination therapy was tried, such as a cisplatin and irinotecan regimen. During the follow-up period, eight patients (7/12, 66.7%) had developed multiple liver, brain, lung and bone metastases and died within 24 months. Figure 2 shows treatment strategies that have been organized from these collective cases to extract potential valuable information.

### Patient survival

**Figure 3:**
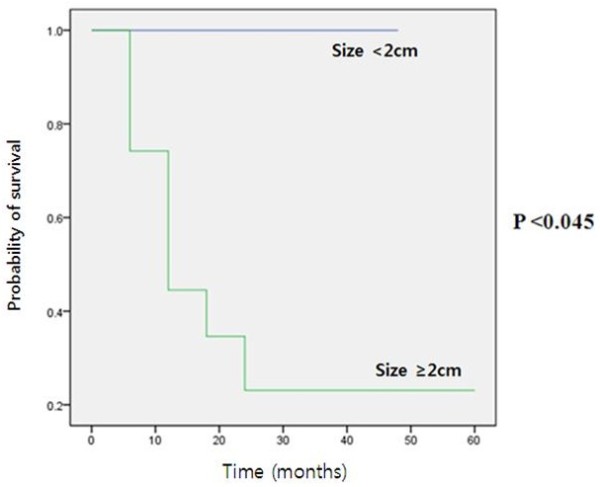
**Kaplan-Meier survival curve for patients with esophageal neuroendocrine tumors based on tumor size.** Patients with tumors less than 2.0 cm in size appeared to have significantly better survival compared with those that had tumors greater than 2.0 cm in size.

With respect to the final survival rate, 15 out of 26 patients were still alive, and nine patients had died. Two remaining patients were either transferred to other hospitals or were lost during follow-up, thus their final condition was not recorded. The median survival time was 27.04 ± t17.16 months (1–59 months). According to Kaplan-Meier survival analysis, only tumor size (more than 2.0 cm) showed prognostic significance (*P* = 0.045) (Figure 3). Other factors, including tumor location (*P* = 0.345), regional lymph node metastasis (*P* = 0.597), lymphovascular invasion (*P* = 0.096), synaptophysin expression (*P* = 0.155) and chromogranin expression (*P* = 0.557), were not significantly associated with survival rate.

## Discussion

To our knowledge, this study, in which we have presented 26 cases of esophageal NETs, is to date the largest study of its kind with long-term follow-up. According to our data, the incidence of esophageal NETs was 1.3% (26/2,037). The data in Korea show slightly higher rates compared with Western countries [8]. This may be due to genetic and racial differences, but can also be correlated with the development and widespread use of screening endoscopy [1].

Esophageal NETs are usually known to occur in males, and we also found that males were predominant in our study (male to female ratio of 4:1) [10]. In our study, all of them occurred sporadically; that is, they were not found to be of a familial type, such as type 1 multiple endocrine neoplasia (MEN 1) or neurofibromatosis type 1 [11]. Dysphagia was the most common presenting symptom and only one patient had a typical carcinoid symptom.

From endoscopic findings, esophageal NETs were typically a single lesion and commonly developed in the lower third of the esophagus. This is because neuroendocrine cells are mainly distributed in mucosal glands of the distal esophagus [6, 12, 13]. In 1990, Attar *et al.* noted a predominant distribution of neuroendocrine carcinomas in the lower third of the esophagus, similar to our results, and related this to the abundance of endocrine cells in this region [12, 13].

Like other malignant tumors, prognosis of esophageal NETs is influenced by the degree of lymph node metastasis [2, 13, 14]. In this study, regional lymph node metastasis or widespread metastasis of primary esophageal NETs was 57.7% (15 out of 26 cases) at diagnosis. This finding underscored that a very high percentage of esophageal NETs were already metastatic at the time of diagnosis, compared with NETs that occurred at other anatomical locations [15, 16].

There are no data on prognostic factors associated with esophageal NETs. Kaplan-Meier survival analysis done in this study showed that size was an important prognostic factor. A tumor size of more than 2.0 cm appeared to be a prognostic factor for poor survival and widespread metastasis of esophageal NETs. However, all other parameters, as well as regional lymph node metastasis and lymphovascular invasion, did not have prognostic significance in this study. The lack of regional lymph node metastasis and lymphovascular invasion appeared to predict better survival, but it was not statistically significant. This was because chemotherapy affected patient survival, which improved during the study period [17–20].

In previous studies, inconsistent results were presented on whether synaptophysin or chromogranin expression was associated with better prognosis. One Korean multicenter study reported that synaptophysin and chromogranin expression were not significant prognostic factors [1]. However, only in appendix NETs was synaptophysin associated with a better prognosis [1]. In this study, we performed survival analysis for esophageal NETs, but synaptophysin and chromogranin were not prognostic factors.

In addition, proliferative activity, assessed by mitotic count or Ki-67 immunostaining, was previously described as a significant prognostic factor in a previous study [21]. However, the mitotic index was recorded in only 20% of collected pathology reports and the Ki-67 labeling index in only 10% of collected pathology reports because most pathologic reports were made prior to the 2010 WHO classification. Thus, we did not perform survival analysis and multivariate analysis associated with the mitotic or Ki-67 index.

Treatment of primary NETs depends on clinical staging [2, 22]. However, currently, a specific treatment algorithm for esophageal NETs has not been established. Three of the patients were treated using endoscopic resection with a clear margin and there were no recurrences. Endoscopic treatment was performed only on patients with tumors less than 1.0 cm (range, 0.2–0.8 cm) and they did not have regional lymph node metastasis. Six patients were treated by surgical resection only, or combined chemotherapy. Tumor size was more than 1.0 cm (range 1.4–1.7 cm) in all six cases, and only one patient died because of a post-operative complication. Twelve patients with regional and distant metastasis were treated with only palliative chemotherapy. The most frequent combination used was a cisplatin and etoposide regimen. This combination may have resulted in most of the tumor progression seen in up to two-thirds of patients. During the follow-up period, eight patients developed widespread metastases and died within 24 months after the diagnosis. Therefore, there was a very limited role for chemotherapy in the treatment of advanced esophageal NETs, which did not show a benign clinical course [13, 23, 24]. In Figure 2, algorithms are shown for the management of esophageal NETs. Esophageal NETs, estimated endoscopically to be <1.0 cm in diameter without lymph node metastasis, were rarely metastatic. Therefore, these tumors were considered good candidates for endoscopic resection. However, most of the esophageal NETs were already showing lymph node or widespread metastasis at the time of diagnosis, and were associated with a poor prognosis in spite of systemic chemotherapy.

In addition, about half of the patients had poorly differentiated neuroendocrine tumors (PEDCs) or small cell carcinomas with respect to the 2000 WHO classification. This was in regard to tumors of more than 1.0 cm in diameter, commonly of the ulcerative type, and there was early involvement of the esophageal wall, or it spread to regional lymph nodes in the initial invasion of adjacent organs. In our data on esophageal NETs, the 1-year survival rates of well-differentiated endocrine tumors was 100%, in contrast to 85% for well-differentiated endocrine carcinomas and 33% for poorly differentiated endocrine carcinomas.

The limitations of this study were as follows: First, different pathologists at each institution diagnosed esophageal NETs. Because the international consensus has not been achieved for histologic classification of esophageal NETs, some pathologists did not mention their histology type. Second, the 2000 WHO classification system was mainly used in this study. This is because most of the pathologic reports were made before the 2010 WHO classification. However, to our knowledge, this study regarding the 26 cases of esophageal NETs was the largest with a long-term follow-up period that was sufficient to provide valuable clinical information.

## Conclusions

Although the general incidence of gastrointestinal NETs is sharply increasing, the incidence of esophageal NETs is still very rare. Unlike other NETs that progress with a benign course, most of the esophageal NETs were already showing metastasis at the time of diagnosis, were rapidly proceeding, and were associated with a poor prognosis. However, when there was no lymphatic metastasis, the size of the tumor was less than 1.0 cm, and the NETs were not pathologically poorly differentiated, the tumor was completely removable via an endoscopic method and there was no recurrence during the follow-up period.
